# Supplementary material for: The interactome of intact mitochondria by cross-linking mass spectrometry provides evidence for coexisting respiratory supercomplexes
Source: Mol Cell Proteomics. 2017 Dec 8;17(2):216–32. doi: 10.1074/mcp.RA117.000470 (PMC5795388; doi:10.1074/mcp.RA117.000470)
Supplement: Supplemental Data [file supp_RA117.000470_133922_0_supp_23978_fzffwf.zip › spectra_annotation/mito_spectra_annotation/394-1-27-1-14-1.pdf]

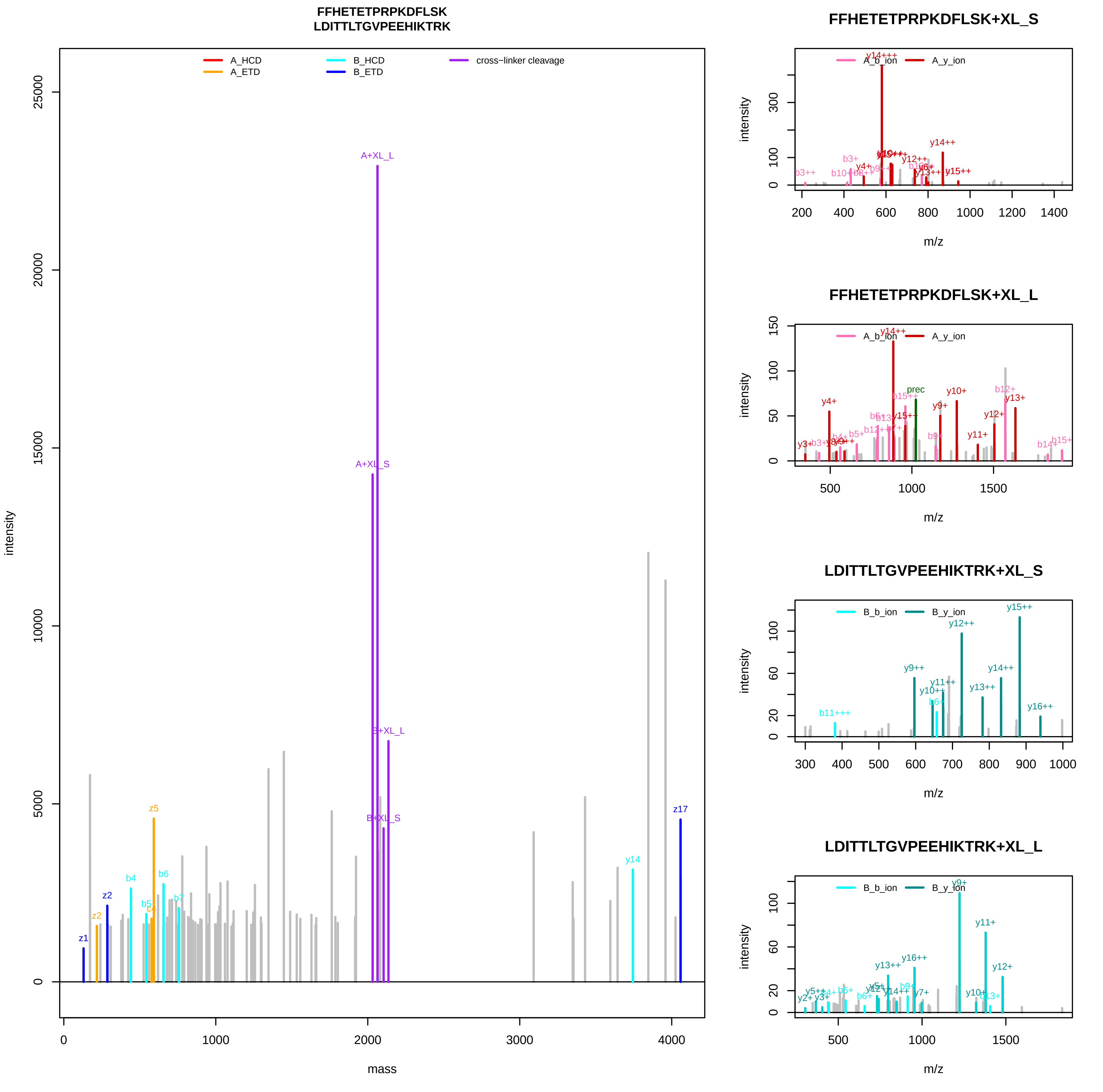
FFHETETPRPKDFLSK
LDITTLTGVPEEHIKTRK
A_HCD
A_ETD
B_HCD
B_ETD
cross−linker cleavage
intensity
mass
0
5000
10000
15000
20000
25000
0
1000
2000
3000
4000
A+XL_L
A+XL_S
B+XL_L
B+XL_S
z1
z2
z2
b4
b5
c4
z5
b6
b7
y14
z17
FFHETETPRPKDFLSK+XL_S
A_b_ion
A_y_ion
intensity
m/z
0
100
300
200
400
600
800
1000
1200
1400
FFHETETPRPKDFLSK+XL_L
A_b_ion
A_y_ion
intensity
m/z
0
50
100
150
500
1000
1500
prec
LDITTLTGVPEEHIKTRK+XL_S
B_b_ion
B_y_ion
intensity
m/z
0
20
60
100
300
400
500
600
700
800
900
1000
LDITTLTGVPEEHIKTRK+XL_L
B_b_ion
B_y_ion
intensity
m/z
0
20
60
100
500
1000
1500
